# COLEC10: A potential tumor suppressor and prognostic biomarker in hepatocellular carcinoma through modulation of EMT and PI3K-AKT pathways

**DOI:** 10.1515/biol-2022-0988

**Published:** 2025-02-26

**Authors:** Rui-Sheng Ke, Yun Dai, Yan-ling Tu, Zhao-Hui Liu, Kun-Zhai Huang, Fu-Xing Zhang

**Affiliations:** Department of General Surgery, The First Affiliated Hospital of Xiamen University, School of Medicine, Xiamen University, Xiamen 361003, Fujian, China; Endoscopic Diagnosis and Treatment Department, The First Affiliated Hospital of Xiamen University, School of Medicine, Xiamen University, Xiamen, 361003, Fujian, China; Department of Neurology, Zhongshan Hospital Affiliated to Xiamen University, Xiamen, 361001, China; Department of General Surgery, The First Affiliated Hospital of Xiamen University, School of Medicine, Xiamen University, No. 55, Zhenhai Road, Siming District, Xiamen 361003, Fujian, China

**Keywords:** HCC, COLEC10, EMT, Hedgehog signaling, PI3K-AKT pathway

## Abstract

Hepatocellular carcinoma (HCC) is a cancer with poor prognosis, underscoring the urgent need for enhanced detection and management. This study aimed to investigate the role of Collectin Subfamily Member 10 (COLEC10) in HCC, which was revealed to be associated with various diseases. Bioinformatics tools, including GEO, cBioPortal, and TCGA, were used to identify differentially expressed genes. The prognostic significance of COLEC10 was assessed in two patient cohorts, and its functional impact on Hep3B and SMMC7721 cells was evaluated through CCK-8 and Transwell assays. The underlying mechanisms of COLEC10 in HCC progression were explored using flow cytometry and western blot. COLEC10 was downregulated in HCC and associated with poorer overall survival and disease progression. The potential interaction of COLEC10, CCBE1, and FCN3 was predicted. COLEC10, CCBE1, and FCN3 were identified as prognostic indicators for HCC. Overexpression of COLEC10 inhibited the proliferation, migration, and invasion of HCC cells. COLEC10 overexpression induced G0/G1 cell cycle arrest and suppressed epithelial–mesenchymal transition (EMT), COLEC10 regulated protein expression in the Hedgehog pathway and phosphorylation of key proteins in the PI3K-AKT pathway. COLEC10 is an independent prognostic factor of HCC. COLEC10 regulates EMT, Hedgehog, and PI3K-AKT pathways, providing new ideas for targeted therapy of HCC.

## 1 Introduction

Hepatocellular carcinoma (HCC) is a growing cause of cancer-related death worldwide [1]. The most recent statistical reports indicate that liver cancer is the third leading cause of cancer-related mortality worldwide, with nearly half of all liver cancer cases occurring in China [2]. HCC often develops in individuals with cirrhosis or chronic liver diseases, including chronic hepatitis B or C (HBV or HCV) infection, alcohol-related liver disease, or the increasingly common steatogenic liver disease associated with metabolic dysfunction [3]. However, HCC can still only be diagnosed at an advanced stage, complicating the treatment efforts. In addition, we lack strong, predictable, and prognostic biomarkers to tailor treatment strategies, which make it difficult to determine which patients will benefit most from a particular treatment. This highlights the urgent need to acquire molecular data on HCC to improve the development of biomarkers for improved detection and management [1].

Collectin subfamily member 10 (COLEC10), located on chromosome 8q23-q24.1, belongs to the C-lectin family. The majority of C-lectins typically serve as cell surface receptors within the innate immune system [4]. Unlike other members of the C-lectin family, which are secreted proteins, COLEC10’s gene product is a cytoplasmic protein. This unique feature suggests that COLEC10 may serve distinct biological functions compared to other C-lectins [5,6]. Genetic polymorphisms within the COLEC10 gene have been linked to the etiology of the 3-methylcholanthrene syndrome [7]. In HCC, COLEC10 expression is significantly downregulated, and its reduced expression levels are indicative of a poor prognosis [8]. It has been reported that miR-452-5p regulates HCC cell proliferation and metastasis by targeting COLEC10, leading to malignant progression of HCC [9]. Despite these findings, the precise molecular mechanisms underlying COLEC10’s role in HCC progression remain to be fully understood. We aim to elucidate COLEC10’s function in HCC by integrating bioinformatics analysis with experimental validation, potentially revealing novel targets for HCC diagnosis and therapeutic intervention.

## 2 Methods and materials

### 2.1 Gene expression profile data and identification of differentially expressed genes (DEGs) in HCC

The Gene Expression Omnibus (GEO) databases offer a valuable resource for bioinformatic analysis of gene expression profiles implicated in the tumorigenesis of a spectrum of human cancers. We accessed the gene expression profile datasets GSE62232, GSE107170, GSE98383, GSE74656, and GSE14520 from the National Center for Biotechnology Information (NCBI) GEO repository (https://www.ncbi.nlm.nih.gov/geo/). The platforms for these datasets were as follows: GPL570 (Affymetrix Human Genome U133 Plus 2.0 Array) for GSE62232, GSE107170, and GSE98383; and GPL16043 (GeneChip® PrimeViewTM Human Gene Expression Array) for GSE74656. Additionally, we evaluated COLEC10 expression in HCC and adjacent non-cancerous tissues from patients with HBV-related HCC using the NCBI GEO dataset (accession: GSE14520) [10]. This dataset contained 445 samples from HCC patients, comprising 247 tumor tissues and 241 non-cancerous tissues across platforms GPL571 (Affymetrix Human Genome U133A 2.0 Array) and GPL3921 (Affymetrix HT Human Genome U133A Array). The downloaded data were analyzed using GEO2R to identify DEGs between tumor and adjacent normal samples within each GEO profile, based on the respective microarray platform. Downregulated DEGs were identified using a fold-change (FC) threshold of log2FC ≤ −1.1 and a *P*-value cutoff of <0.05. Venny 2.1.0 online software (http://bioinfogp.cnb.csic.es/tools/venny/index.html) was employed to analyze and validate the overlapping DEGs among the four datasets. The candidate genes that were down-expressed in HCC samples were considered as the cohort of DEGs.

### 2.2 Data and methods for specific bioinformatics analysis of COLEC10 in HCC

In the present study, HCC-related datasets were retrieved from the GEO and The Cancer Genome Atlas (TCGA) databases. These datasets were subsequently integrated and subjected to a comprehensive bioinformatics analysis. To elucidate the functional roles of key genes, Kyoto Encyclopedia of Genes and Genomes (KEGG) and Gene Ontology (GO) enrichment analyses were conducted. We utilized the DAVID Bioinformatics Resources version 6.7 for conducting KEGG pathway and GO biological processes enrichment analyses [11]. A *P*-value threshold of less than 0.05 was set to filter significant results. Additionally, we performed gene set enrichment analysis (GSEA) through LinkedOmics to further analyze the enrichment of these genes in KEGG pathways, with a false discovery rate (FDR) *q*-value threshold of less than 0.05 for significance [12]. GEPIA, an online tool, analyzed RNA-Seq data from TCGA and GTEx to investigate COLEC10 expression profiles across human tissues and cancers. GEPIA and UCSC Xena project were also utilized to examine COLEC10 expression patterns in liver hepatocellular carcinoma (LIHC) stages and subtypes. UALCAN analyzed COLEC10 expression in relation to HCC survival outcomes [13]. Kaplan–Meier Plotter was used to plot survival curves for these genes in HCC patients [14]. Lastly, the HCCDB database provided an integrative molecular view of HCC, illustrating the co-expression network of COLEC10 and offering regression analysis of gene expression and patient survival data across various studies.

### 2.3 Clinical prognostic value of COLEC10 expression in HBV-related HCC

To assess the correlation between candidate biomarkers and the clinicopathological features of HCC patients, we analyzed the association between COLEC10 expression and the clinicopathological characteristics of 371 HCC patients using the LinkedOmics database. The FDR was determined using the Benjamini-Hochberg (BH) method. This study focused on the clinical prognostic value of COLEC10 expression in HCC associated with HBV. HBV-associated HCC samples from the GSE14520 dataset were included as cohort 1 for the analysis of COLEC10 expression levels in tissues. Variables of interest were tested and transformed within Cox proportional hazards regression models to assess their impact. Survival analysis was conducted to evaluate the clinical outcomes of patients with HBV-related HCC, with overall survival (OS) and relapse-free survival (RFS) defined as per previously published methodologies [15]. Tumor specimens from 149 HBV-positive HCC patients admitted to the First Affiliated Hospital of Xiamen University from May 2019 to July 2020 constituted Cohort 2, and the final follow-up time was May 1, 2024. Assessments of liver function, tumor differentiation, tumor staging according to the TNM classification system, criteria for defining curative resection, and patient inclusion criteria were performed as previously described [15]. All pathological data were originally evaluated by two independent pathologists. Pathological specimens from 149 HCC tissue samples were sectioned into 4 μm slices for immunohistochemical analysis, and staining scores were determined using the methods outlined in previous studies [15].

### 2.4 Immunohistochemistry (IHC) analyses

In a study, 149 HCC tissues were processed for IHC analysis. These tissue specimens were obtained from HCC patients who were treated at the First Affiliated Hospital of Xiamen University (i.e., Cohort 2). These specimens were collected by the Department of Pathology of our hospital during the patients' surgical procedures. Sections (4 μm thick) were mounted on slides and underwent deparaffinization and rehydration. Antigen retrieval was performed using EDTA buffer and heating. Endogenous peroxidase was quenched with 3% H_2_O_2_. Sections were incubated with primary antibodies (COLEC10, GNMT, and KMO) overnight at 4°C, followed by horseradish peroxidase (HRP)-conjugated secondary antibodies. The EnVision kit was used for detection, and staining was visualized with 3,3′-diaminobenzidine and hematoxylin. Sections were dehydrated and mounted. Staining was scored by blinded pathologists, with negative samples having <10% stained cells and positive samples categorized based on staining percentage and intensity.


**Informed consent:** Informed consent has been obtained from all individuals included in this study.
**Ethical approval:** The research related to human use has been complied with all the relevant national regulations, institutional policies and in accordance with the tenets of the Helsinki Declaration, and has been approved by the First Affiliated Hospital of Xiamen University (China).

### 2.5 Signature of COLEC10, CCBE1, and FCN3 in HCC

Prognostic models were established to assess the prognostic impact of COLEC10, CCBE1, and FCN3 genes in HCC using TCGA RNA-sequencing data. TPM normalization and log2 transformation were applied to the data, ensuring inclusion of samples with complete clinical profiles. The log-rank test identified survival differences among groups, while TimeROC analysis evaluated the predictive accuracy of the gene signature and risk score. LASSO regression with 10-fold cross-validation was used for feature selection via the R package glmnet [16]. Regularization, feature selection, and adjusting model complexity were used to balance bias and variance to improve the predictive performance of the LASSO Cox regression model. A multivariate Cox regression analysis constructed the prognostic model using the survival package. Kaplan–Meier curves were plotted, and statistical significance was determined from log-rank tests, univariate Cox regression, and *P*-values (<0.05) [17]. All analyses were conducted in R (version 4.0.3).

### 2.6 Cell culture

The hepatic normal epithelial cell line (THLE-2) and a panel of HCC cell lines, including PLC/PRF/5, SMMC7721, Hep3B, HepG2, and Huh-7, were procured from BeNa Culture Collection (Beijing, China). Among these, PLC/PRF/5 and Hep3B are cell lines derived from HBV-positive HCC cases, whereas HepG2, SMMC7721, and Huh-7 are HBV-negative HCC cell lines. Routine cell culture was performed using Dulbecco’s Modified Eagle Medium (DMEM) (HyClone, USA) at a temperature of 37°C in an atmosphere containing 5% CO_2_. The growth medium was supplemented with fetal bovine serum (FBS) (Gibco, USA) at a concentration of 10% and a penicillin/streptomycin mixture (HyClone, USA) at a concentration of 1%.

### 2.7 Cell transfection

Well-grown cells, with a confluence of approximately 70–80%, were passaged into six-well plates and cultured overnight. Transfection was initiated once the cells re-entered the logarithmic growth phase. The growth medium was replaced with antibiotic-free DMEM, followed by the transfection of the COLEC10 overexpression plasmid (oe-COLEC10) and its corresponding empty vector control (Ctrl) into the HCC cells using Lipofectamine 2000 (Thermo Fisher Scientific, USA). Six hours post-transfection, the antibiotic-free medium was aspirated and replaced with complete DMEM, after which the cells were allowed to continue culturing. The oe-COLEC10 plasmid was synthesized by cloning the full-length COLEC10 coding sequence into the pcDNA3.1 expression vector, while the control vector was pcDNA3.1 alone.

### 2.8 Quantitative real-time PCR (qRT-PCR)

Total RNA was extracted using the Ultrapure RNA Kit (Cwbio, Jiangsu, China), according to the manufacturer’s protocol. Complementary DNA (cDNA) was synthesized via reverse transcription with the HiFiScript cDNA Synthesis Kit (Cwbio, Jiangsu, China), utilizing the extracted RNA as a template. Subsequent amplification reactions were conducted on an ABI 7500 qPCR instrument (Thermo Fisher Scientific, USA), employing cDNA as a template and following the guidelines provided with the SYBR Premix Ex Taq Kit (Takara, Dalian, China). The relative expression level of COLEC10 was quantified using the 2^−ΔΔCt^ method, with glyceraldehyde 3-phosphate dehydrogenase (GAPDH) serving as an endogenous control.

### 2.9 Cell proliferation assay

Hep3B and SMMC7721 cells, transfected with the oe-COLEC10 plasmid, were seeded into 96-well culture plates, with triplicate wells for each experimental group. Subsequently, 10 μL of the CCK-8 reagent (Solarbio, Beijing, China) was mixed with 90 μL of DMEM to prepare the working solution. The culture medium for the cells was refreshed with this working solution every 24 h. One hour following the medium change, absorbance at 450 nm (OD450) was measured using an enzyme-linked immunosorbent assay reader (BioTek, USA). This process was repeated for a total of four consecutive measurements, after which the growth curves of the cells were plotted for analysis.

### 2.10 Transwell assay

The migratory and invasive capabilities of Hep3B and SMMC7721 cells transfected with oe-COLEC10 were assessed using Transwell assays. Cells were trypsinized and resuspended in FBS-free medium, then aliquoted into the upper chamber of a Transwell insert (Millipore, USA) at a density of 5 × 10^4^ cells per 200 μL. The lower chamber was filled with 500 μL of complete medium. Following a 24 h incubation period, non-migrated cells remaining on the upper surface of the membrane were gently removed with a cotton swab. The cells that had migrated to the lower surface were fixed with a 4% paraformaldehyde solution for 20 min, and then stained with 0.1% crystal violet solution for 15 min. Subsequently, the number of migrated cells was determined by counting five randomly selected fields under a light microscope and the data were recorded. For the invasion assay, the upper chamber of the Transwell was precoated with a layer of Matrigel (Corning, USA) and incubated for 30 min to allow gelation. The subsequent steps were identical to those of the migration experiment.

### 2.11 Cell cycle assay

The cell cycle distribution of Hep3B and SMMC7721 cells transfected with oe-COLEC10 was evaluated using a flow cytometry-based assay. Following transfection, cells were collected by trypsinization, washed with phosphate-buffered saline (PBS), and then resuspended in PBS containing 95% ethanol for fixation. The cells were fixed overnight at −20°C. On the subsequent day, cells were washed twice with PBS to remove the ethanol and then incubated with a propidium iodide staining solution for 20 min in the dark. After staining, the cells were analyzed for their cell cycle phase distribution using a flow cytometer (BD Biosciences, USA).

### 2.12 Western blot

Transfected HCC cells were lysed using a radio-immunoprecipitation assay buffer containing 1% protease inhibitor cocktail. The cells were agitated on a shaker at 4°C for 30 min to ensure thorough lysis, followed by centrifugation to collect the supernatant. The protein concentration in the supernatant was determined using a bicinchoninic acid assay. Equal amounts of protein samples were then resolved by sodium dodecyl sulfate-polyacrylamide gel electrophoresis. Subsequently, the resolved proteins were electrotransferred onto polyvinylidene fluoride (PVDF) membranes (Millipore, USA). The membranes were blocked with 5% skimmed milk for 2 h to prevent non-specific antibody binding. After blocking, the membranes were incubated with primary antibodies overnight at 4°C with gentle agitation. The COLEC10 antibody (Novus, USA) used in this study was diluted at 1:1,000. Other antibodies used in this study were N-cadherin (#13116T), E-cadherin (#14472S), Vimentin (#5741T), GLI1 (#3538T), SHH (#2207T), PTCH1 (#2468T), SMO (#92981S), phospho-AKT (#2920S), PI3K (#4257), AKT (#4060T), and phospho-PI3K (#4228T) from Cell Signaling Technology, all diluted at a ratio of 1:1,000, and GAPDH (#60004-1-Ig, Proteintech) diluted at a ratio of 1:3,000. Following the incubation with primary antibodies, the PVDF membranes were washed three times with Tris-buffered saline containing 0.1% Tween 20 (TBST). The membranes were then incubated with a HRP-conjugated secondary antibody (Thermo Fisher Scientific, USA) diluted at a ratio of 1:5,000 for 1 h at room temperature. After further five washes with TBST, the membranes were developed for visualization of the immunoreactive bands.

### 2.13 Statistical analysis

Statistics and visualization of data using SPSS software (version 21.0, SPSS Inc., Chicago, IL) and GraphPad Prism 9.0 software. Unpaired *t*-test was used for comparison between groups. *χ* ^2^ test was used to analyze the relationship between genes and clinicopathological features of patients. The Kaplan–Meier method was used to draw survival curves, and the log-rank test was used to compare the differences between the curves. Multivariate Cox regression was used to analyze the predictive value of genes for the prognosis of HCC patients. Correlation of gene expression was analyzed using spearman’s correlation coefficient. Differences were considered significant when the result with a *P*-value was <0.05.

## 3 Results

### 3.1 Identification and analysis of DEGs in HCC

**Figure 1:**
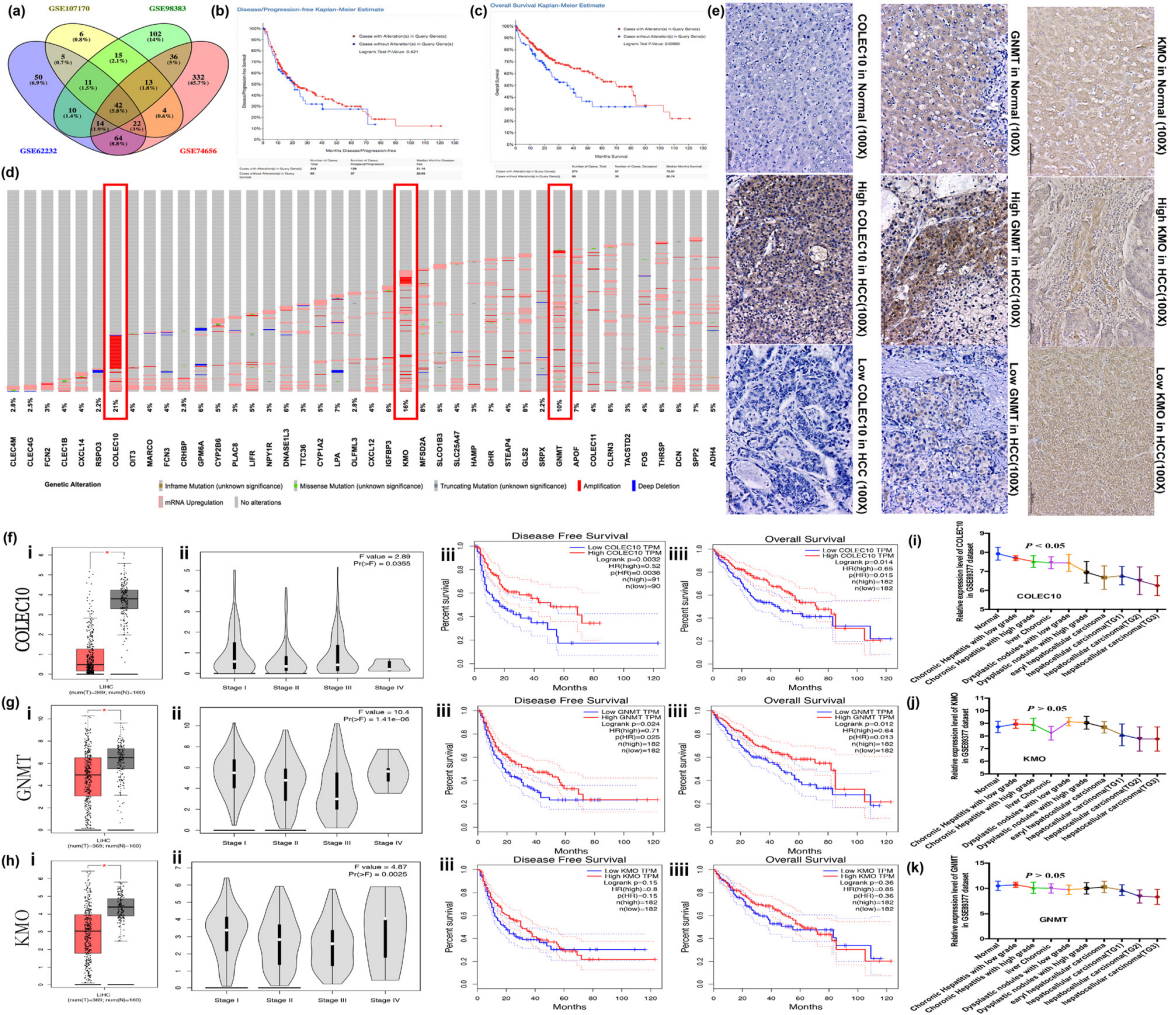
Identification and analysis of DEGs in HCC. (a) Venn diagram depicting the intersection of downregulated genes across the GSE62232, GSE107170, GSE98383, and GSE74656 profiles, as analyzed by the Venny 2.1.0 software. A total of 42 DEGs downregulated in all four datasets were selected. (b) and (c) Kaplan–Meier survival analysis depicting DFS (b) and OS (c) in HCC patients with and without alterations in the 42 DEGs. (d) Oncoprint representation of genetic alterations and expression levels of the 42 DEGs in liver cancer tissue. (e) Representative immunohistochemical image showing high and low expression levels of COLEC10, KMO, and GNMT in tumor tissue from a patient with HCC (100× magnification). (f)–(h) Correlation between mRNA levels of COLEC10 (f), KMO (g), and GNMT (h) with tumor stage and prognosis in HCC patients was assessed using data from the GEPIA database. (i)–(k) The GSE89377 dataset was utilized to verify the association between gene expression levels of COLEC10 (i), KMO (j), and GNMT (k) and the nine stages of liver cancer progression associated with viral hepatitis.

Data retrieved from the GEO repository were analyzed using the GEO2R tool to identify DEGs between tumor and adjacent normal samples within each GEO profile, based on the specific microarray platform utilized. Downregulated DEGs were identified by calculating the FC in gene expression, applying a threshold criterion of log2FC ≤ −1.8 and a *P* value <0.05 for DEG selection. log2FC ≤ −1.8 indicated downregulation of gene expression, which was determined based on empirical and actual data distribution, and *P* < 0.05 denoted a statistically significant difference. According to these criteria, we identified 1,106 genes that were downregulated in HCC tissues compared to non-cancerous samples. As depicted in Figure 1a, an intersectional analysis of the GSE62232, GSE107170, GSE98383, and GSE74656 datasets revealed 42 genes that were consistently downregulated across these datasets. We further investigated the genetic alterations of these 42 DEGs within a cohort of 440 HCC cases available in the cBioPortal database. The presence of genetic alterations in these 42 DEGs in HCC tissues was not significantly associated with disease-free survival (DFS) in HCC patients (*P* = 0.421, Figure 1b), but were significantly associated with poorer OS (*P* = 0.009, Figure 1c). Additionally, the analysis indicated that 76% (275/440) of HCC cases within this cohort displayed alterations in these 42 DEGs (Figure 1d). Enrichment analysis was performed for these 42 downregulated genes, as summarized in Table 1. Data from cBioPortal confirmed that the top three genes with the highest frequency of alterations in HCC tissues were COLEC10, KMO, and GNMT, with 21, 16, and 10% of cases exhibiting alterations, respectively (Figure 1d). COLEC10, in particular, emerged as a promising candidate that may play a critical role in the progression of HCC.

**Table 1:** Presentation of part of the GO and KEGG pathway enrichment analysis for the downregulated DEGs

Category	Term	Term	*P*-value	Benjamini
GOTERM_BP_FAT	GO:0034097	Response to cytokine stimulus	1.30 × 10^−2^	1.00 × 10
GOTERM_BP_FAT	GO:0019748	Secondary metabolic process	1.30 × 10^−2^	1.00 × 10
GOTERM_BP_FAT	GO:0001867	Complement activation, lectin pathway	1.30 × 10^−2^	9.80 × 10^−1^
GOTERM_BP_FAT	GO:0006955	Immune response	1.70 × 10^−2^	9.60 × 10^−1^
GOTERM_BP_FAT	GO:0006790	Sulfur metabolic process	2.70 × 10^−2^	9.50 × 10^−1^
GOTERM_BP_FAT	GO:0007166	Cell surface receptor linked signal transduction	4.50 × 10^−2^	9.90 × 10^−1^
GOTERM_BP_FAT	GO:0006952	Defense response	4.50 × 10^−2^	9.80 × 10^−1^
KEGG_PATHWAY	hsa00830	Retinol metabolism	8.00 × 10^−3^	2.10 × 10^−1^
KEGG_PATHWAY	hsa00980	Metabolism of xenobiotics by cytochrome P450	9.80 × 10^−3^	1.40 × 10^−1^
KEGG_PATHWAY	hsa00982	Drug metabolism	1.00 × 10^−2^	1.00 × 10^−1^
KEGG_PATHWAY	hsa04060	Cytokine–cytokine receptor interaction	2.60 × 10^−2^	1.80 × 10^−1^

Immunohistochemical analysis of clinical hepatoma tissue samples revealed that COLEC10, KMO, and GNMT proteins were predominantly localized to the cytoplasm of hepatoma cells in all samples (Figure 1e). The expression levels of COLEC10, KMO, and GNMT were observed to be markedly reduced in tumor tissues compared to matched normal counterparts (*P* < 0.05, Figure 1f–h). The expression patterns of COLEC10, KMO, and GNMT in LIHC were correlated with tumor staging (P(NF) < 0.05) (Figure 1f–h). Additionally, the mRNA levels of COLEC10 and GNMT were found to be associated with RFS and OS in HCC patients, as analyzed using data from GEPIA (*P* < 0.05, Figure 1f and g). To further investigate the correlation between gene expression and liver cancer progression, we utilized the GSE89377 dataset. Our analysis indicated that low COLEC10 expression in tumors showed a significant inverse correlation with disease progression (*P* < 0.05, Figure 1i). In contrast, the expression levels of KMO and GNMT in tumors did not show a significant association with disease progression (*P* > 0.05, Figure 1j and k). Collectively, these findings suggest that COLEC10 may play a pivotal role in the malignant progression of HCC.

### 3.2 Analysis of COLEC10-co-expressed genes, protein–protein interaction (PPI) network, and prognostic significance in HCC

**Figure 2:**
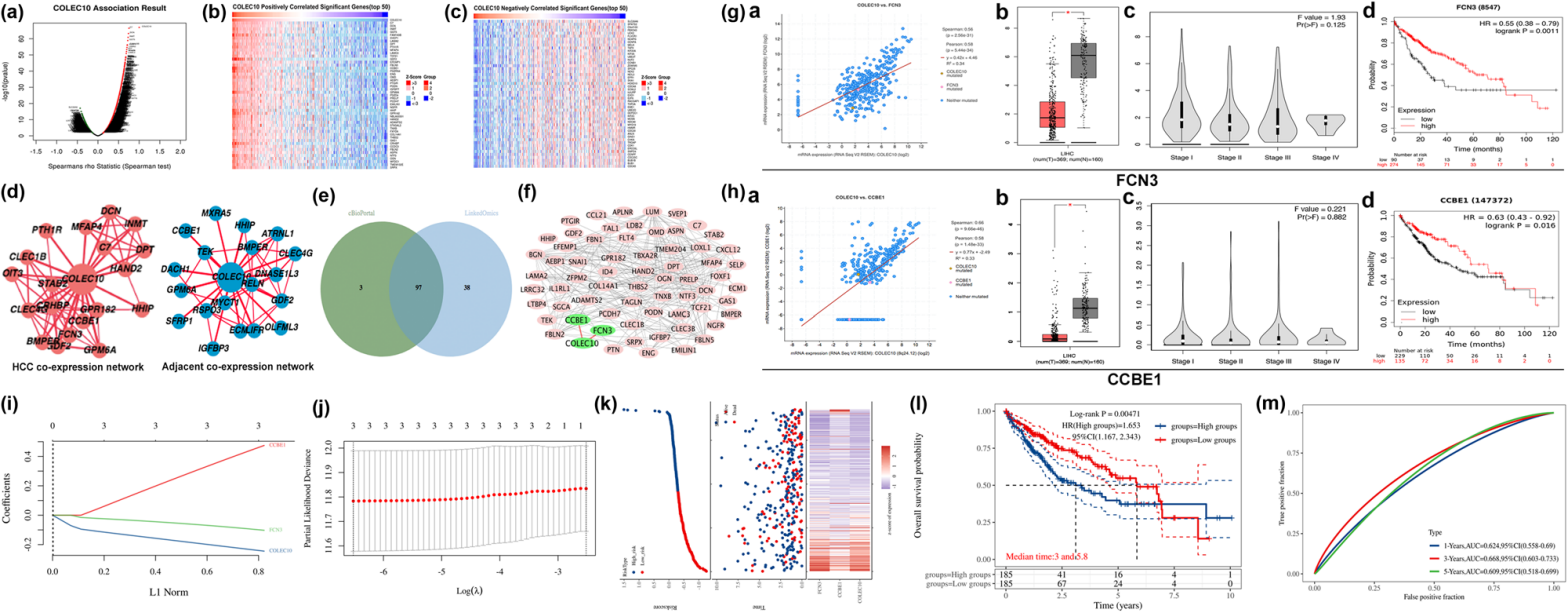
Analysis of COLEC10-co-expressed genes, PPI network, and prognostic significance in HCC. (a) Funnel chart illustrating significant target genes positively/negatively correlated with COLEC10 (*P* < 0.001). (b) Top 50 genes positively correlated with COLEC10. (c) Top 50 genes negatively correlated with COLEC10. (d) PPI network analysis of COLEC10 protein in liver cancer tissue and paracancerous tissue, constructed using the HCCDB database. (e) Selection of 97 genes with a Spearman’s correlation coefficient >0.55 as COLEC10 co-expressed genes overlapping in “cBioPortal” and “LinkedOmics”; (f) the PPI network for COLEC10 was visualized using Cytoscape, with an enrichment *P*-value <1.0 × 10^−16^. (g) and (h) Evaluation of the relationship between CCBE1 and FCN3 mRNA levels and tumor stage and prognosis in HCC patients. (i) Coefficients of selected features as determined by the lambda parameter in the LASSO Cox regression model. (j) Plot of partial likelihood deviance versus log (*λ*) for the LASSO Cox regression model. (k) Representation of the Riskscore, survival time, and survival status for the selected dataset; the top scatter plot illustrates the distribution of Riskscores from low to high, with different colors denoting different groups, and the scatter plot distribution correlates Riskscores of different samples with survival time and status. Bottom heatmap displays gene expression patterns from the signature. (l) Kaplan–Meier survival curves for the two stratified groups based on Riskscore. (m) ROC curves demonstrating the predictive accuracy of the survival model in HCC, with AUC at 1-year, 3-year, and 5-year intervals (1-year AUC = 0.624, 3-year AUC = 0.668, 5-year AUC = 0.609).

Utilizing the LinkedOmics database, we identified 9,220 genes associated with COLEC10 expression through co-expression analysis (*P* < 0.05). The top 50 genes with the highest positive and negative Spearman correlation coefficients with COLEC10 expression were visualized as heatmaps (Figure 2a–c). Subsequent analysis, as detailed in Table 2, demonstrated that COLEC10 expression significantly correlated with tumor purity, pathologic stage, pathology T-stage, pathology N-stage, and OS in HCC patients within the LinkedOmics database. Analysis via the HCCDB database demonstrated alterations in the PPI network of COLEC10 between HCC tissues and their adjacent non-tumor tissues (Figure 2d). A selection of 97 genes with a Spearman’s correlation greater than 0.55 were identified as co-expressed with COLEC10 across both the cBioPortal and LinkedOmics databases (Figure 2e). These 97 genes were employed to construct a PPI network using the STRING database, which showed significant enrichment (PPI enrichment *P*-value <1.0 × 10^−16^), and the network was visualized using Cytoscape. Notably, the PPI analysis indicated potential interactions between COLEC10 and the proteins CCBE1 and FCN3 (Figure 2f), with significant correlations observed between the expression levels of these genes and COLEC10 (*P* < 0.001, Figure 2Ga and Ha). Nevertheless, the interactions between these genes need to be further verified experimentally. Expression levels of FCN3 and CCBE1 were found to be significantly reduced in tumor tissues compared to their non-tumor matched normal counterparts from the GTEx database (Figure 2Gb and Hb). However, their expression in LIHC was not associated with tumor stage (Pr(NF) > 0.05, Figure 2Gc and Hc). Furthermore, the mRNA levels of FCN3 and CCBE1 were associated with OS in HCC patients (Figure 2Gd and Hd).

**Table 2:** Association of COLEC10 expression with clinical features of HCC patients in the LinkedOmics database

Query	Statistic	*P*-value	FDR (BH)
years_to_birth (Spearman correlation)	−1.91 × 10^−2^	7.29 × 10^−1^	8.02 × 10^−1^
Tumor_purity (Spearman correlation)	−2.48 × 10^−1^	4.17 × 10^−6^	4.59 × 10^−5^
pathologic_stage (Kruskal–Wallis test)	1.19 × 10^1^	7.60 × 10^−3^	2.09 × 10^−2^
pathology_T_stage (Kruskal–Wallis test)	1.23 × 10^1^	6.51 × 10^−3^	2.09 × 10^−2^
pathology_N_stage (Wilcox test)	−9.40 × 10^−1^	3.31 × 10^−2^	7.27 × 10^−2^
pathology_M_stage (Wilcox test)	1.88 × 10^−1^	8.11 × 10^−1^	8.11 × 10^−1^
radiation_therapy (Wilcox test)	−2.99 × 10^−1^	2.88 × 10^−1^	5.28 × 10^−1^
residual_tumor (Kruskal–Wallis test)	1.58 × 10	4.53 × 10^−1^	6.23 × 10^−1^
race (Kruskal–Wallis test)	1.46 × 10	6.91 × 10^−1^	8.02 × 10^−1^
ethnicity (Wilcox test)	−2.71 × 10^−1^	4.27 × 10^−1^	6.23 × 10^−1^
overall_survival (Cox regression test)	−1.21 × 10^−1^	2.24 × 10^−3^	1.23 × 10^−2^

Notes: Query – gene/site/protein in given target dataset (dataset with association was performed). Signal strength – estimate/coefficient/statistic obtained from respective statistical method used for analysis; *P*-value – *P*-value obtained from statistical method; FDR (BH) – FDR is calculated by BH method.

The LASSO Cox regression model, which is used to identify key prognostic factors while avoiding overfitting, was applied to construct prognostic gene signature [16]. The LASSO Cox regression model was applied to analyze COLEC10, CCBE1, and FCN3 as prognostic indicators. The optimal *λ* (lambda) value was chosen based on the smallest median of the sum of squared residuals, identifying three potential predictors (Figure 2i and j). COLEC10, CCBE1, and FCN3 were confirmed as prognostic factors for HCC. A risk score calculation for these three genes was performed for subsequent univariate and multivariate Cox regression analyses. A scatter plot was generated to illustrate the distribution of the risk score across different samples in relation to survival time and status, with different colors representing distinct groups (Figure 2k). The calculated risk score formula was Riskscore = (−0.2444)*COLEC10 + (0.4743)*CCBE1 + (−0.1026)*FCN3, with lambda.min = 0.0015. Patients were stratified into high-risk and low-risk groups based on the median expression levels of the three candidate genes. The low-risk group consistently exhibited a better prognosis compared to the high-risk group. Survival curves were plotted using the Kaplan–Meier method (Figure 2l). Additionally, the prognostic efficiency of these risk factors was compared through receiver operating characteristic (ROC) curves. The results demonstrated that the ROC curves for the survival model in HCC were as follows: 1-year area under the curve (AUC) = 62.4%, 3-year AUC = 66.8%, and 5-year AUC = 60.9% (Figure 2m), suggesting that these three candidate genes have potential utility as prognostic biomarkers in HCC.

### 3.3 Two patient cohorts verified the correlation between COLEC10 expression and clinical prognosis of HCC

**Figure 3:**
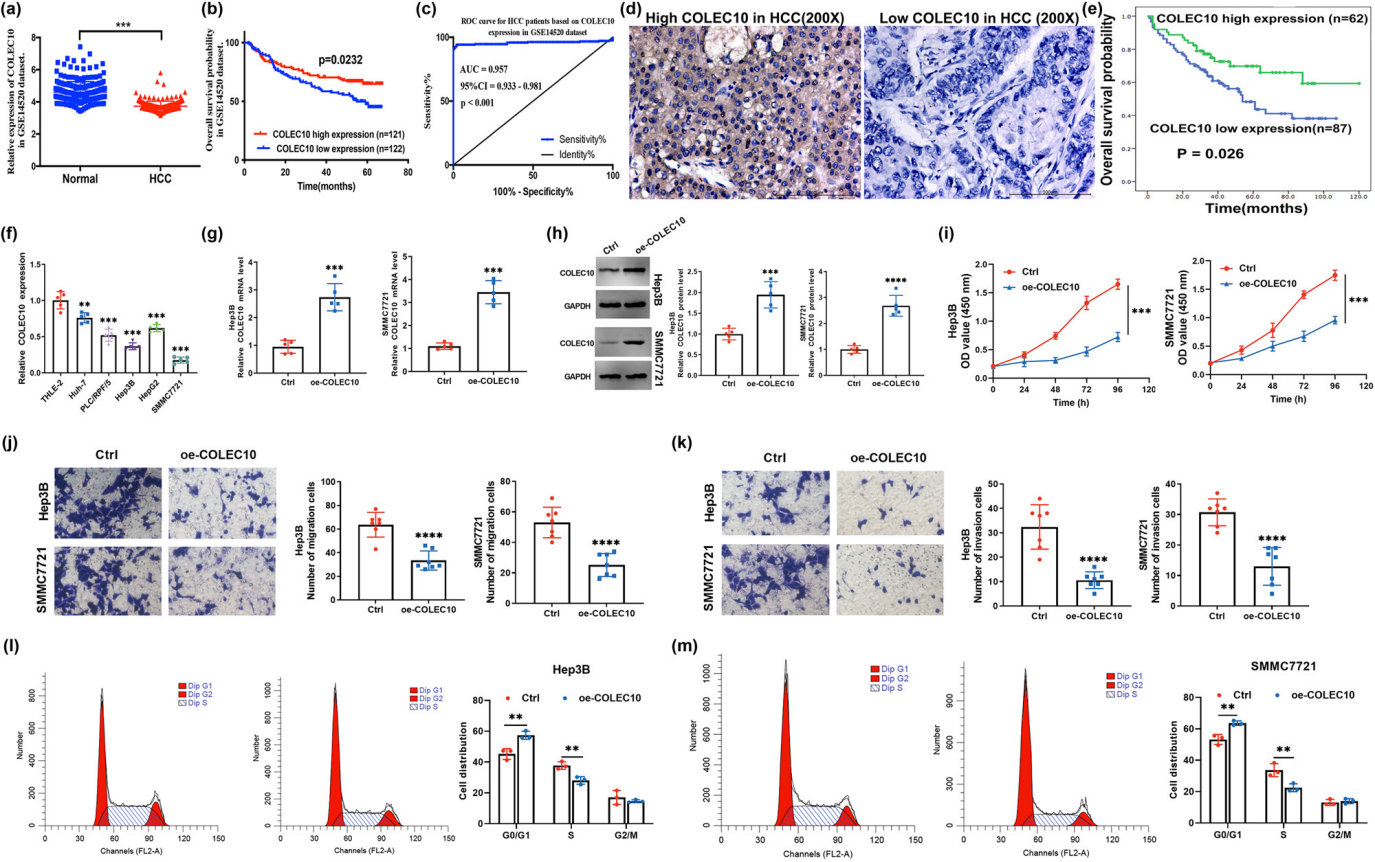
Correlation between COLEC10 expression and clinical outcomes in HCC patients and the impact of COLEC10 on cellular processes. (a) COLEC10 mRNA expression is downregulated in HCC tissues (*n* = 247) compared to non-HCC tissues (*n* = 241) in the GSE14520 dataset. (b) Patients with low COLEC10 expression exhibit significantly poorer OS in the GSE14520 cohort (*P* < 0.05). (c) ROC curve analysis for HCC patient stratification based on COLEC10 expression levels, with an AUC of 0.957, a cutoff value of 2.800, a sensitivity of 90.2%, and a specificity of 90.9%. (d) Representative immunohistochemical images of high and low COLEC10 expression in HCC tumor tissue (100× magnification). (e) Kaplan–Meier curves depicting differences in OS among HCC patients; low COLEC10 expression is associated with poorer OS (*P* < 0.05). (f) Relative COLEC10 mRNA levels in a panel of HCC cell lines, including Huh-7, PLC/PRF/5, Hep3B, HepG2, and SMMC7721. (g) and (h) Transfection efficiency assessment of the oe-COLEC10 construct in Hep3B and SMMC7721 cells. (i) Effect of COLEC10 overexpression on the proliferative capacity of Hep3B and SMMC7721 cells. (j) and (k) Impact of COLEC10 overexpression on the migratory and invasive capabilities of Hep3B and SMMC7721 cells. (l) and (m) Influence of COLEC10 on the cell cycle distribution of Hep3B and SMMC7721 cells. Statistical significance is denoted by ***P < 0.001 and ****P < 0.0001.

In the GSE14520 cohort (*n* = 247, referred to as cohort 1), COLEC10 mRNA expression was observed to be significantly lower in HCC tissues compared to non-HCC tissues (Figure 3a). Patients with low COLEC10 expression exhibited significant associations with gender, primary tumor size, and multinodular tumor (Table 3). Concurrently, multivariate analysis established multinodular tumor, cirrhosis, BCLC staging, CLIP staging, and COLEC10 expression as independent prognostic factors for OS in HCC patients (Table 4). Notably, those with low COLEC10 expression faced poorer prognoses, as indicated by OS rates (Figure 3b). The GSE14520 cohort analysis also demonstrated that the prognostic value of COLEC10 expression levels in HCC tissues was highly significant, with a high AUC score of 0.957 in the ROC analysis (Figure 3c). To further substantiate the clinical relevance, immunohistochemical analysis was conducted on a separate cohort of 149 HBV-positive HCC patients (referred to as cohort 2), aiming to delineate the relationship between COLEC10 expression levels and clinicopathological characteristics (Figure 3d). COLEC10 expression significantly correlated with tumor location, TNM staging, serum alpha-fetoprotein (AFP) levels, and survival rates (Table 5). Multifactorial analysis reaffirmed vascular invasion and COLEC10 expression as independent prognostic factors for OS (Table 6). The 5-year OS rate among patients with low COLEC10 expression was markedly lower compared to those with high COLEC10 expression (Figure 3e). Collectively, the findings from these two cohorts underscore the association between COLEC10 expression and disease progression, as well as its utility as a prognostic indicator in HCC patients.

**Table 3:** Associations between COLEC10 (207420_at) expressions with the clinicopathological characteristics of HCC patients in GSE14520

		COLEC10 expression			
Variable	*N*	Low	High	*P* value	Chi-square value
Age (years)					
≤55	159	82	77	0.294	0.458
>55	83	39	44		
Gender					
Female	31	10	21	0.027	4.477
Male	211	111	100		
ALT (U/L)					
≤50	142	70	72	0.448	0.068
>50	100	51	49		
Main tumor size (cm)					
≤5	153	68	85	0.013	5.567
>5	88	53	35		
Multinodular					
No	190	88	102	0.021	4.801
Yes	52	33	19		
Serum AFP level (ng/mL)					
≤300	128	62	66	0.348	0.27
>300	110	57	53		
Cirrhosis					
No	19	10	9	0.5	0.057
Yes	223	111	112		
BCLC staging					
0–A	172	81	91	0.283	0.534
B–C	53	28	25		
CLIP staging					
0	98	43	55	0.142	1.45
1–5	127	66	61		

Note: Chi-square test for all the other analyses. *P* values less than 0.05 were considered statistically significant. Boldface type indicates significant values. Abbreviations: AFP, alpha-fetoprotein; ALT, alanine aminotransferase; BCLC, Barcelona clinic liver cancer. There are missing data in the table, as follows: *BCLC staging, *CLIP staging are missing 17 cases of data, respectively; ^Main tumor size group missing 1 case data; #Serum AFP level group missing 4 cases of data.

**Table 4:** Cox regression analysis of OS in HCC patients (242 cases, GSE14520 database)

		Univariate analysis		Multivariate analysis	
Variables		HR (95% CI)	*P*	HR (95% CI)	*P*
Age (year)	≤55 vs >55	0.731(0.470–1.135)	0.163		
Gender	Male vs female	1.858(0.901–3.833)	0.094		
Main tumor size (cm)	≤5 vs >5	1.960(1.309–2.933)	0.001	1.038(0.609–1.767)	0.892
ALT (U/L)	≤50 vs >50	1.155(0.772–1.727)	0.483		
Multinodular	No vs yes	1.653(1.064–2.569)	0.025	0.313(0.156–0.628)	0.001
Serum AFP level (ng/mL)	≤300 vs >300	1.686(1.126–2.527)	0.011	0.739(0.386–1.415)	0.362
Cirrhosis	No vs yes	5.093(1.255–20.671)	0.023	4.236(1.037–17.312)	0.044
BCLC staging	0–A vs B–C	2.194(1.384–3.478）	<0.001	5.133(2.611–10.088)	<0.001
CLIP staging	0 vs 1–5	2.609(1.775–3.835)	<0.001	2.462(1.129–5.366)	0.023
COLEC10	Low vs high	0.627(0.418–0.942)	0.025	0.636(0.407–0.992)	**0.046**

Cox proportional hazards regression. Abbreviations: OS, overall survival; HR, hazard ratio; CI, confidential interval; AFP, alpha-fetoprotein; ALT, alanine aminotransferase; BCLC, Barcelona clinic liver cancer; CLIP, Cancer of the Liver Italian Program. There are missing data in the table, as follows: *TNM stage, *BCLC staging, *CLIP staging are missing 17 cases of data, respectively; ^Main tumor size group missing 1 case data; #Serum AFP level group missing 4 cases of data.

**Table 5:** Correlation between COLEC10 expression and clinicopathological characteristics in HBV-positive HCC patients (*n* = 149)

			COLEC10 level		
Characteristics		*N*	Low (*n*)	High (*n*)	*P* value
Age (year)	≤55	82	52	30	0.113
	>55	67	35	32	
Gender	Male	138	80	58	0.487
	Female	11	7	4	
Tumor location	Left	30	13	17	0.049
	Right	119	74	45	
TNM stage	I/II	103	55	48	0.046
	IIIa	45	32	14	
Tumor size (cm)	≤5	83	48	35	0.505
	>5	66	39	27	
Vascular invasion	No	58	31	27	0.21
	Yes	91	56	35	
Serum AFP level (μg/L)	≤400	98	50	48	0.009
	>400	51	37	14	
Tumor encapsulation	no	51	30	21	0.54
	yes	98	7	41	
HBV DNA load (IU/mL)	≤104	63	32	31	0.75
	>104	86	55	31	
Survival	Alive	63	44	19	0.012
	Dead	86	43	43	

Abbreviations: TNM, tumor-nodes-metastasis; AFP, alpha-fetoprotein; HBV, Hepatitis B virus. Chi-square test for all analyses. *P* values less than 0.05 were considered statistically significant.

**Table 6:** Univariate and multivariate Cox regression analysis of OS in HBV-positive HCC patients (*n* = 149)

Characteristics		Univariate analysis	*P*-value	Multivariate analysis	*P* value
		HR (95% CI)		HR (95% CI)	
Age (year)	≤55 vs >55	1.091(0.665–1.790)	0.73		
Gender	Male vs female	0.697(0.253–1.913)	0.486		
Tumor location	Left vs right	1.344(0.678–2.624)	0.389		
TNM stage	I/II vs IIIa	2.402(1.458–3.957)	<0.001	1.481(0.741–2.958)	0.266
Tumor size (cm)	≤5 vs >5	2.255(1.364–3.729)	0.002	1.467(0.0.721–2.987)	0.29
Vascular invasion	No vs yes	7.178(3.5347–14.527)	<0.001	6.051(2.823–12.968)	<0.001
Serum AFP level (μg/L)	≤400 vs >400	1.471(0.883–2.449)	0.138		
Tumor encapsulation	No vs yes	2.449(0.1.413–4.244)	0.001	0.879(0.512–1.509)	0.639
HBV DNA load (IU/mL)	>104 vs ≤104	2.354(1.372–4.037)	0.002	1.672(0.947–2.950)	0.076
COLEC10	Low vs high	0.549(0.320–0.940)	0.029	1.777(1.032–3.061)	0.038

Note: *P* values <0.05 were considered significant. Abbreviations: AFP, alpha-fetoprotein; TNM, tumor, node, metastasis; HR, hazard ratio; CI, confidential interval.

### 3.4 Overexpression of COLEC10 inhibits cellular proliferation, migration, and invasion in HCC cells

The impact of COLEC10 overexpression on HCC cell lines was investigated. Initially, it was observed that COLEC10 mRNA levels were significantly reduced in Huh-7, PLC/PRF/5, Hep3B, HepG2, and SMMC7721 cells, with particularly notable effects in Hep3B and SMMC7721 cells (Figure 3f). Given these findings, both Hep3B and SMMC7721 cells were selected for further examination of the role and mechanisms of COLEC10 in HCC. The oe-COLEC10 vector effectively increased COLEC10 mRNA and protein levels in these cells, demonstrating high transfection efficiency (Figure 3g and Figure S1). Following transfection with oe-COLEC10, the proliferative capacity of Hep3B and SMMC7721 cells was monitored over time, revealing a clear decline in the oe-COLEC10-transfected group compared to the control (Ctrl) group (Figure 3h).

The effect of COLEC10 overexpression on cell migration was assessed using Transwell assays. Both Hep3B and SMMC7721 cells exhibited a significantly reduced number of migrated cells in the oe-COLEC10 group as opposed to the Ctrl group (Figure 3i). Additionally, the invasive capacity of these cells was evaluated, with results indicating fewer invasive cells in the oe-COLEC10 group (Figure 3j). To determine if COLEC10 overexpression could affect cell cycle progression, flow cytometry analysis was performed. A comparison of cell distribution across G0/G1, G2/M, and S phases revealed a significant increase in G0/G1-phase cells and a corresponding decrease in S-phase cells in the oe-COLEC10 group (Figure 3k and l). These results suggest that COLEC10 overexpression may induce G0/G1 cell cycle arrest, thereby potentially suppressing cell cycle progression.

### 3.5 COLEC10 overexpression suppresses epithelial–mesenchymal transition (EMT) and regulates the Hedgehog pathway in HCC cells

**Figure 4:**
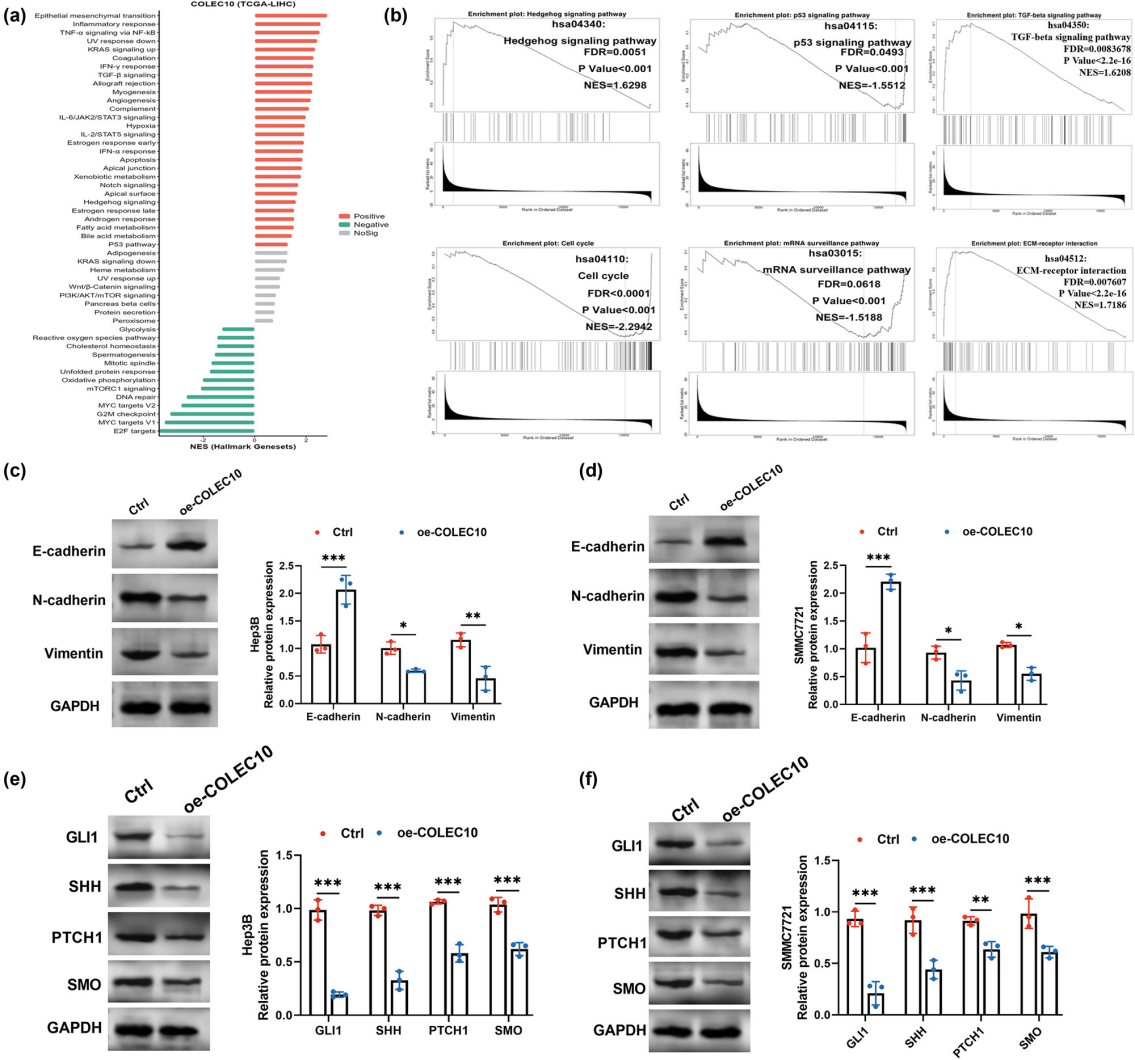
Impact of COLEC10 overexpression on EMT and the Hedgehog pathway in HCC cells. (a) Hallmark enrichment analysis results of COLEC10 expression in the TCGA-HCC dataset, indicating the involvement of COLEC10 in various biological processes and pathways. (b) Utilization of the LinkedOmics database to examine the correlation of COLEC10 with the Hedgehog signaling pathway, P53 signaling pathway, TGF-β signaling pathway, cell cycle regulation, mRNA surveillance pathway, and extracellular matrix receptor interaction. (c) and (d) Detection of EMT-related protein levels to evaluate the role of COLEC10 in the EMT process in Hep3B (c) and SMMC7721 (d) cells. Overexpression of COLEC10 significantly modulated the expression of EMT markers. (e) and (f) Assessment of the effect of COLEC10 overexpression on the levels of key proteins in the Hedgehog pathway in Hep3B (e) and SMMC7721 (f) cells, suggesting COLEC10’s potential regulatory effect on this pathway. *P < 0.05, **P < 0.01, ***P < 0.001.

EMT is a critical process in cancer metastasis, and its regulation by COLEC10 in HCC cells was investigated. Hallmark enrichment analysis of COLEC10-expressing TCGA-HCC samples revealed significant associations with pathways including EMT, Hedgehog signaling, Notch signaling, TGF-β signaling, and the P53 signaling pathway (Figure 4a). Further KEGG enrichment analysis utilizing the LinkedOmics database corroborated the involvement of COLEC10 with the Hedgehog signaling pathway, P53 signaling pathway, TGF-β signaling pathway, and cell cycle regulation (Figure 4b).

To experimentally ascertain the role of COLEC10 in EMT, we overexpressed COLEC10 in HCC cells and assessed the protein expression levels of EMT markers. The overexpression of COLEC10 led to a significant increase in the protein expression of E-cadherin, a marker of epithelial cells, and a concurrent significant decrease in the protein expression of N-cadherin and Vimentin, which are indicative of mesenchymal cells (Figure S2a and b). These findings imply that COLEC10 may play a role in the progression of EMT in HCC.

Western blot analysis was employed to evaluate the impact of COLEC10 overexpression on key proteins within the Hedgehog signaling pathway. In Hep3B cells, a significant downregulation was observed in the expression levels of GLI1, SHH, PTCH1, and SMO in the COLEC10-overexpressing group (Figure S2c). A similar trend was observed in SMMC7721 cells (Figure S2d). Collectively, these results suggest that COLEC10 may act as a regulator of the Hedgehog signaling pathway in HCC cells.

### 3.6 COLEC10 engagement in the PI3K-AKT signaling pathway in HCC

**Figure 5:**
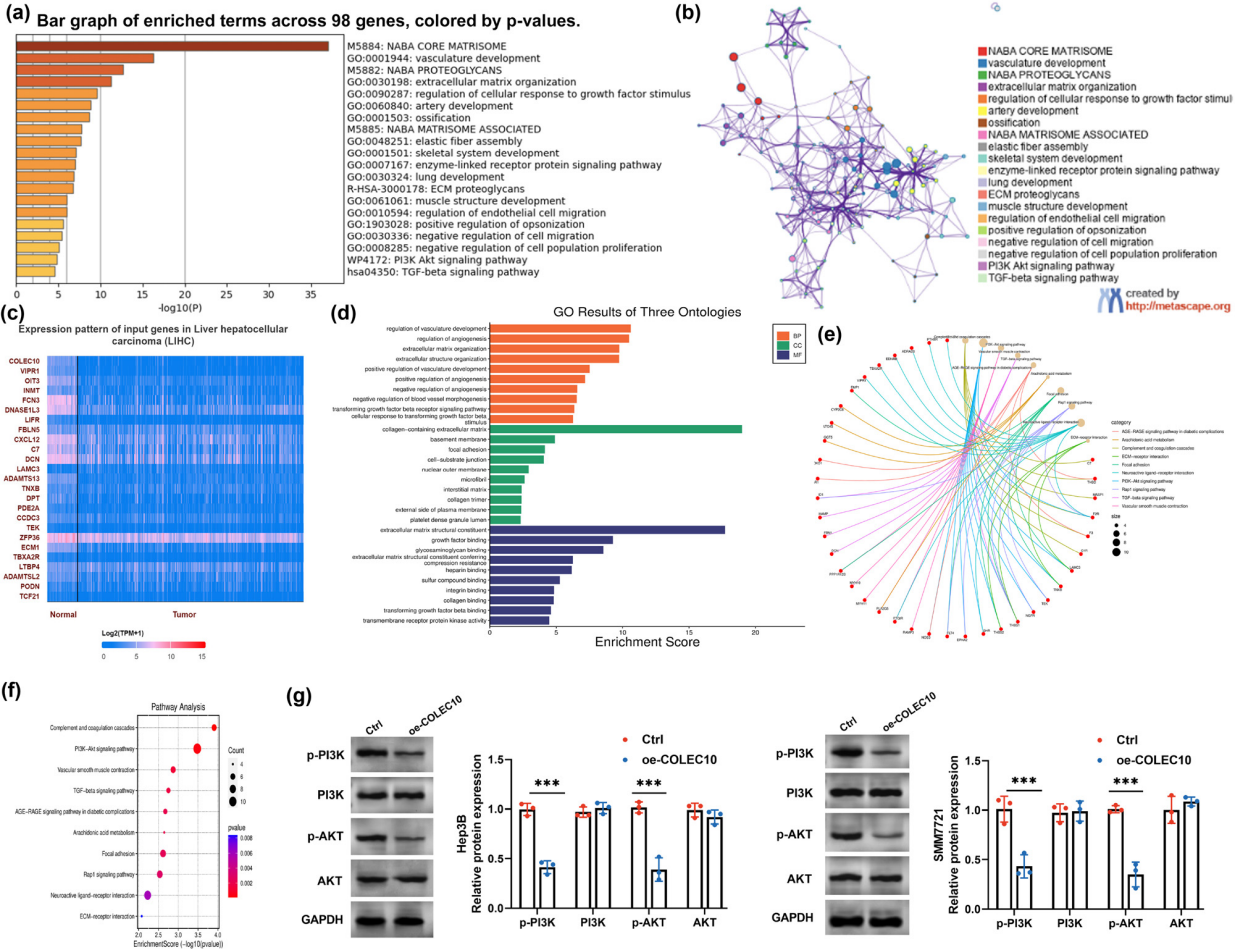
COLEC10 engages with the PI3K-AKT signaling pathway in HCC. (a) Functional and pathway enrichment analysis of 98 co-expressed genes, inclusive of COLEC10, was conducted using the Metscape database. (b) Visualization of the PPI network, organized by distinct functional modules. (c) Utilization of the UALCAN database to identify genes that are positively correlated with COLEC10 expression in HCC. (d) GO enrichment analysis of the co-expressed genes associated with COLEC10, revealing their molecular functions and biological processes. (e) and (f) Further analysis using KEGG enrichment to delineate the signaling pathways involved in HCC that are enriched for COLEC10-associated genes. (g) Western blot analysis demonstrating that overexpression of oe-COLEC10 significantly reduced the phosphorylation levels of PI3K (p-PI3K) and AKT (p-AKT) in Hep3B and SMMC7721 cells compared to the control (Ctrl) group. ***P < 0.001.

To delineate the signaling pathways associated with COLEC10, a functional enrichment analysis was conducted on 98 genes, including COLEC10, utilizing the Metscape database. This analysis included 97 genes with a Spearman correlation coefficient greater than 0.55, which were identified from screenings in both the cBioPortal and LinkedOmics databases. The results highlighted the involvement of the PI3K-AKT signaling pathway, TGF-β signaling pathway, and pathways related to the negative regulation of cell migration and proliferation (Figure 5a). A visual PPI network was constructed, delineating distinct functional modules (Figure 5b). Subsequently, the UALCAN database was employed to identify genes positively correlated with COLEC10 in HCC. This analysis revealed 161 genes, including VIPR1, OIT3, INMT, and FCN3, which demonstrated positive associations with COLEC10 in HCC (Figure 5c). GO enrichment analysis of these genes indicated that COLEC10 is involved in the molecular functions of extracellular matrix components and is associated with the regulation of angiogenesis and vascular development (Figure 5d). Further KEGG enrichment analysis confirmed the involvement of the PI3K-AKT signaling pathway (Figure 5e and f). Based on these findings, we hypothesize that COLEC10 may contribute to HCC progression through the modulation of the PI3K-AKT signaling pathway. To test this hypothesis, western blot analysis was performed to assess the effects of COLEC10 overexpression on key proteins within the PI3K-AKT signaling cascade in HCC cell lines. Overexpression of COLEC10, denoted as oe-COLEC10, was introduced into Hep3B and SMMC7721 cells. The results showed that oe-COLEC10 significantly reduced the expression levels of p-PI3K and p-AKT compared to the control group (Figure S3). Collectively, these data suggest that COLEC10 may play a role in the progression of HCC by regulating the PI3K-AKT signaling pathway.

## 4 Discussion

The molecular mechanisms underlying the progression of HCC have garnered increasing attention in recent decades [18,19]. The C-lectin family, extensively studied in a myriad of diseases including HCC, has shed light on potential therapeutic targets [20–22]. Notably, COLEC12 has been recognized as a key gene involved in the immunosuppression of HCC through bioinformatics analysis [23]. Previous research identified COLEC10 as a hub gene in HCC with potential clinical significance, as revealed by weighted gene co-expression network analysis [24]. The most recent study offers a novel perspective, suggesting that COLEC10 may play a role in modulating endoplasmic reticulum stress signaling in HCC [25].

In the present study, leveraging GEO liver cancer-related expression data, we identified 42 genes that are commonly downregulated in HCC tissues compared to normal tissues, with COLEC10, KMO, and GNMT being the most frequently altered. The cytoplasmic localization of these genes may imply that they play important biological functions in HCC cells, including regulating intracellular signaling pathways or influencing the function of key proteins, suggesting their potential to regulate tumorigenesis and progression. COLEC10, KMO, and GNMT have been demonstrated to regulate several key biological processes in HCC, including cell stemness, metabolism, and apoptosis [26–28]. These proteins may exert an inhibitory effect on HCC progression. Further investigation, particularly of COLEC10, demonstrated its robust association with cancer progression, underscoring its importance as a target for HCC research and potential treatment. Consistent with the findings by Zhang and Wu [8], this study, which included two validation cohorts, showed that low COLEC10 expression in liver cancer tissues correlates with a poor prognosis and is intimately linked to HCC disease progression. This reaffirms the utility of COLEC10 as a risk factor indicator for HCC prognosis. Co-expression analysis identified genes associated with COLEC10 expression. These genes are mostly associated with the development and prognosis of HCC, such as DCN, CCBE1, and NTF3 [29–31]. PPI network analysis implicated CCBE1 and FCN3 as candidates for interaction with COLEC10, with their reduced expressions in tumor tissues and significant correlation with patient survival. CCBE1 has been reported to be downregulated in HCC, preventing tumor progression by promoting mitochondrial fusion. In addition, FCN3 was discovered to regulate ferroptosis sensitivity in HCC cells [30,32]. Therefore, the interaction of COLEC10 with CCBE1 and FCN3 deserves further experimental verification. A LASSO Cox regression model identified COLEC10, CCBE1, and FCN3 as prognostic factors for HCC, facilitating the development of a risk score formula. This formula effectively stratified HCC patients into high-risk and low-risk groups, with the low-risk group exhibiting markedly improved survival outcomes. The predictive efficacy of this risk score system was substantiated by Kaplan–Meier survival analysis and validated through ROC curves. Accordingly, the risk score has stable predictive performance, independent prognostic value, and clinical relevance, and has the potential to be applied as clinical management for patients. In clinical practice, the use of this risk score may facilitate the timely and effective assessment of the risk of poor prognosis in patients, thereby enabling the adjustment and improvement of the therapeutic regimen. This approach may ultimately improve patient prognosis. However, it is imperative to note that this conclusion, drawn from HCC data within TCGA, necessitates further validation through an extensive examination of clinical specimens to substantiate its broader clinical relevance and applicability.

In this study, the prognostic value of COLEC10 in HCC was confirmed through analysis in online databases, and its lower mRNA levels were further substantiated by qRT-PCR in HCC cells. Subsequent *in vitro* experiments demonstrated that COLEC10 overexpression exerted a suppressive effect on HCC cell growth, migration, and invasion, proposing a potential role for COLEC10 as a tumor suppressor in HCC. GSEA conducted on the LinkedOmics database revealed that the Hedgehog signaling pathway, cell cycle, and P53 signaling pathways were significantly enriched in the group with low COLEC10 expression. The cell cycle is a series of highly regulated steps that govern normal cell division, and the ability to sustain unscheduled proliferation is a defining characteristic of cancer [33]. The inactivation of the transcription factor p53 is a common feature across a broad spectrum of tumors [34]. COLEC10 may be implicated in the pathogenesis of HCC through its involvement in the aforementioned signaling pathways.

Dysregulation of the cell cycle can lead to uncontrolled cell proliferation and division, a primary driver in the formation of tumors and a significant factor in cancer development and progression [35]. There is a growing body of evidence suggesting that abnormalities in the cell cycle are associated with the development of HCC [36–38]. The EMT is a complex biological process that plays a crucial role in tumor occurrence and development, particularly in invasion and metastasis [39,40]. Several molecules have been reported to be involved in HCC progression by regulating the EMT process in HCC, including WEE1, c-Myb, and TGF-β1 [41–43]. In this study, the effects of COLEC10 on cell cycle and EMT were verified by *in vitro* experiments based on the results of the bioinformatics analysis. Our findings indicate that overexpression of COLEC10 can induce G0/G1 cell cycle arrest, potentially inhibiting cell cycle progression and, by extension, restraining cell proliferation through the mediation of cell cycle arrest. Furthermore, COLEC10 has been demonstrated to participate in the EMT process by increasing the protein expression of the epithelial marker E-cadherin and decreasing the expression of mesenchymal markers N-cadherin and Vimentin.

The Hedgehog signaling pathway has been implicated in the progression and metastasis of a spectrum of malignancies, including HCC [44,45]. A pivotal role for the Hedgehog pathway in the regulation of the cell cycle has been suggested in the context of HCC [46,47]. Ding et al. demonstrated nonclassical activation of Hedgehog signaling in a mouse model of HCC, which may provide a potential strategy for the treatment of HCC [48]. Additionally, the PI3K-Akt pathway, known for its regulatory influence on cell cycle, proliferation, apoptosis, and metabolism, is a well-characterized signaling mechanism in HCC [49]. In the current study, an integrative approach combining bioinformatics with cellular experimentation was employed. Our findings indicate that the overexpression of COLEC10 leads to a significant downregulation of key proteins within the Hedgehog signaling pathway. We hypothesized that COLEC10 could regulate the transcriptional or translational regulation of these components, but the exact mechanisms need to be further investigated. COLEC10 is also associated with the PI3K-Akt signaling cascade, as evidenced by reduced phosphorylation levels of both PI3K and AKT. These results posit that COLEC10 may exert a critical regulatory function in HCC progression through its influence on EMT, the Hedgehog signaling pathway, and the PI3K-AKT signaling pathway, thereby presenting itself as a promising therapeutic target for HCC.

*In vivo* experiments are essential to study the mechanisms of cancer development. In the future, it will be necessary to verify the effect of COLEC10 on tumor growth *in vivo* through animal experiments, such as the nude mouse subcutaneous transplantation tumor model.

In summary, COLEC10 has been identified as a potential tumor suppressor gene with prognostic implications in HCC. Overexpression of COLEC10 significantly attenuated the malignant phenotype and EMT in HCC cell lines. Our findings suggest that COLEC10 may exert its tumor-suppressive effects, in part, through the modulation of the Hedgehog signaling pathway and the PI3K-AKT signaling cascade, both of which are critical in HCC progression. Given the multifaceted regulatory role of COLEC10, it presents as a promising candidate for the development of targeted therapeutics in HCC.

## Supplementary Material

Supplementary Figure
