# Supplementary material for: COLEC10: A potential tumor suppressor and prognostic biomarker in hepatocellular carcinoma through modulation of EMT and PI3K-AKT pathways
Source: Open Life Sci. 2025 Feb 26;20(1):20220988. doi: 10.1515/biol-2022-0988 (PMC11868708; doi:10.1515/biol-2022-0988)
Supplement: Supplementary Figure [file biol-2022-0988-sm.pdf]

Supplementary material

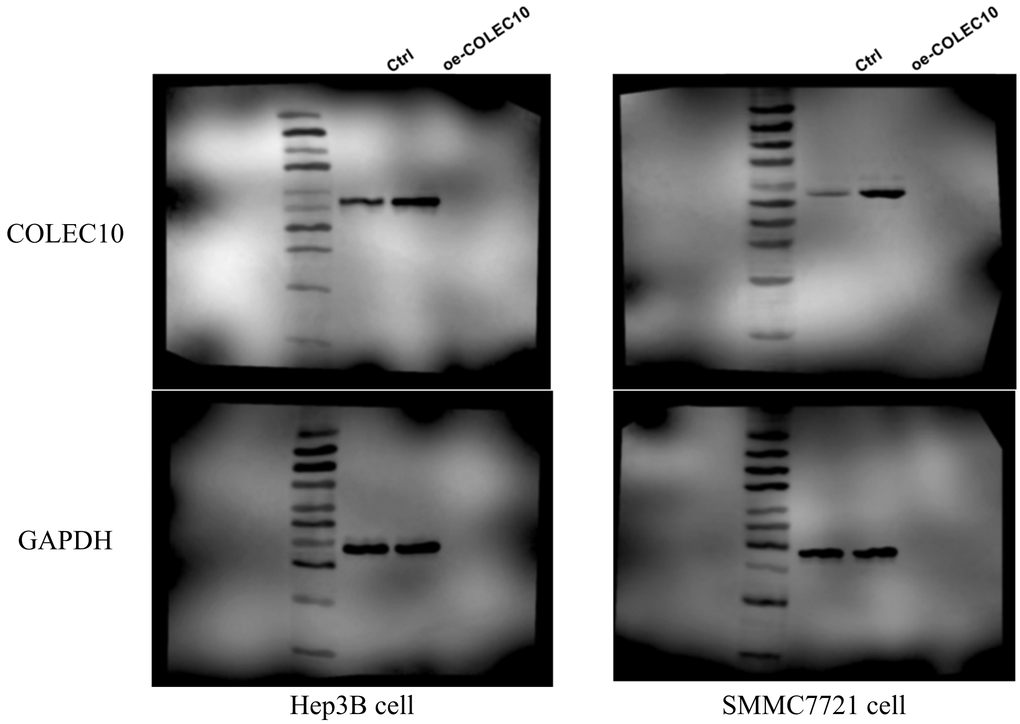

Figure S1: Transfection efficiency assessment of the oe-COLEC10 construct in Hep3B and SMMC7721 cells.

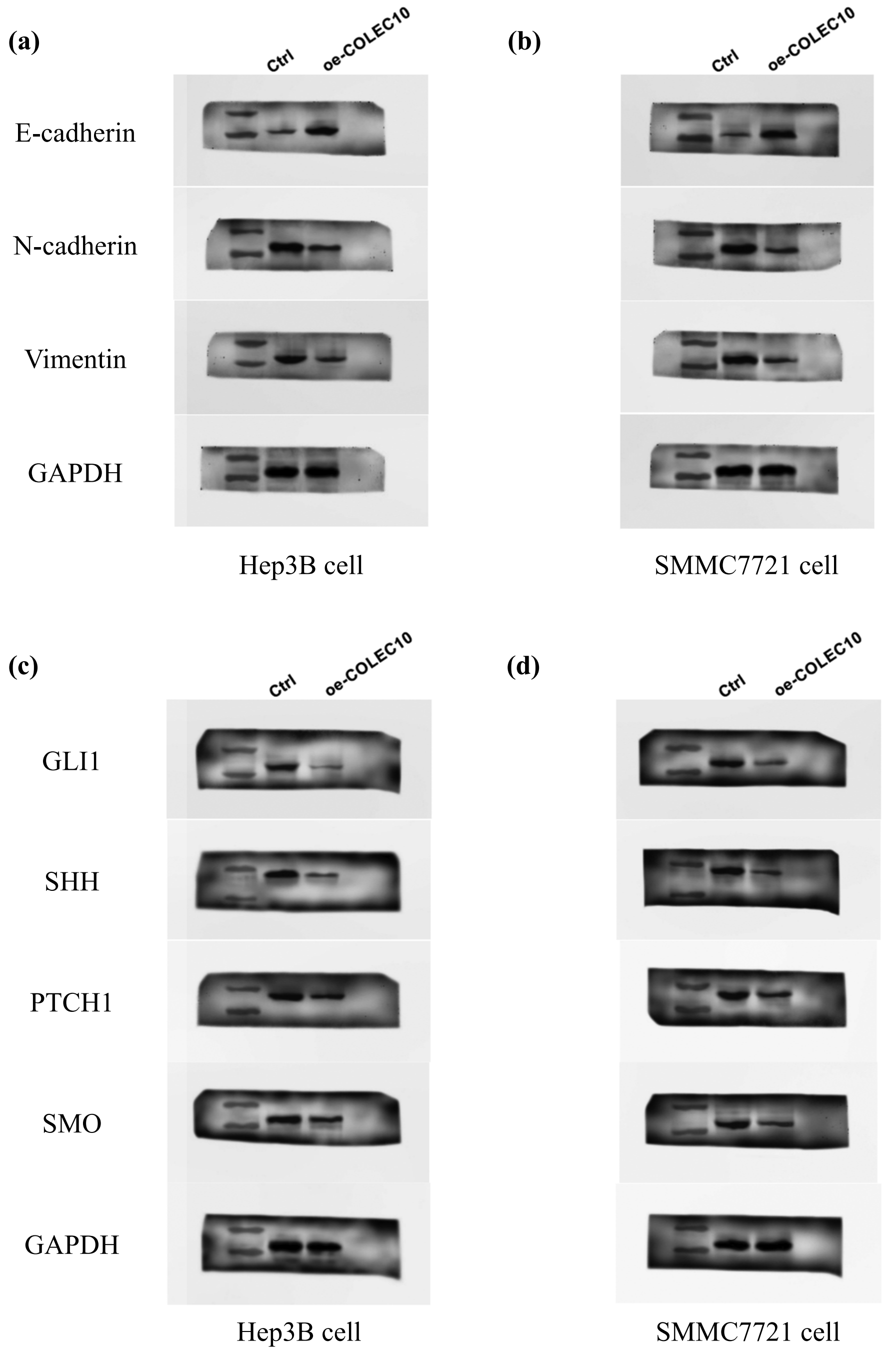

Figure S2: (a) and (b) Detection of EMT-related protein levels to evaluate the role of COLEC10 in the EMT process in Hep3B (a) and SMMC7721 (b) cells. Overexpression of COLEC10 significantly modulated the expression of EMT markers. (c) and (d) Assessment of the effect of COLEC10 overexpression on the levels of key proteins in the Hedgehog pathway in Hep3B (c) and SMMC7721 (d) cells, suggesting COLEC10's potential regulatory effect on this pathway.

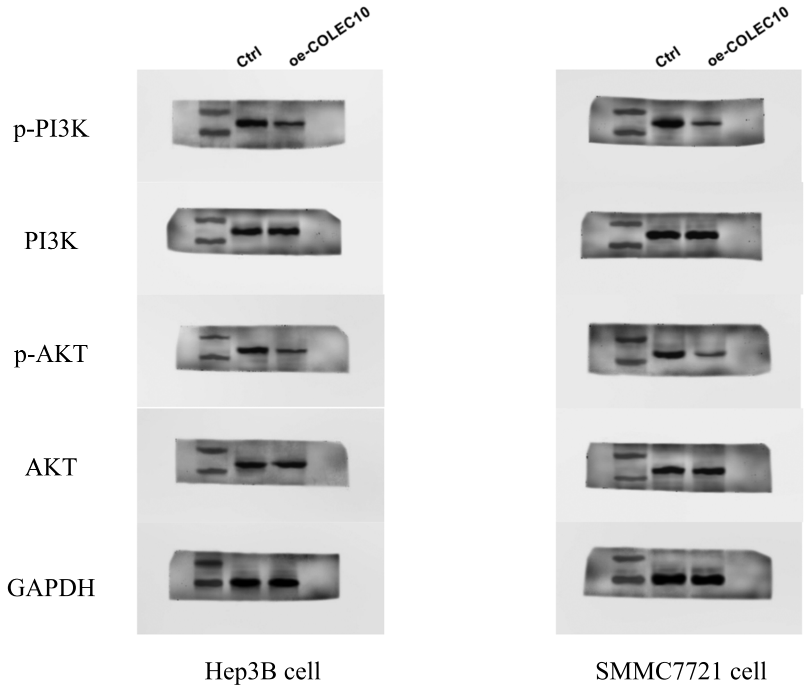

Figure S3: Western blot analysis demonstrating that overexpression of oe-COLEC10 significantly reduced the phosphorylation levels of PI3K (p-PI3K) and AKT (p-AKT) in Hep3B and SMMC7721 cells compared to the control (Ctrl) group.
